# *Helicobacter pylori*: a poor man's gut pathogen?

**DOI:** 10.1186/1757-4749-2-2

**Published:** 2010-03-31

**Authors:** Mohammed Mahdy Khalifa, Radwa Raed Sharaf, Ramy Karam Aziz

**Affiliations:** 1 Department of Microbiology and Immunology, Faculty of Pharmacy, Cairo University, 11562 Cairo, Egypt; 2 Faculty of Pharmacy, Cairo University, 11562 Cairo, Egypt

## Abstract

*Helicobacter pylori *is one of the human pathogens with highest prevalence around the world; yet, its principal mode of transmission remains largely unknown. The role of *H. pylori *in gastric disease and cancer has not been established until the end of the 20^th ^century. Since then, its epidemiology has been extensively studied, and an accruing body of literature suggests that not all humans are equally at risk of infection by this gut pathogen. Here, we briefly review the different epidemiological aspects of *H. pylori *infection with emphasis on those factors related to human poverty. The epidemiology of *H. pylori *infection is characterized by marked differences between developing and developed countries, notably among children. In addition, congruent lines of evidence point out to socioeconomic factors and living standards as main determinants of the age-dependent acquisition rate of *H. pylori*, and consequently its prevalence. These data are alarming in the light of the changing global climate and birth rate, which are expected to change the demography of our planet, putting more children at risk of *H. pylori *and its complications for years to come.

## Introduction

*Helicobacter pylori*, formerly known as *Campylobacter pyloridis *then *Campylobacter pylori*, is one of the human pathogens with highest prevalence around the world; yet, its exact mode of transmission is still uncertain. This organism was isolated from the human stomach but has not been *consistently *isolated from any other niche, and thus the mechanism by which it colonizes the human stomach remains largely unknown.

*H. pylori *is a spiral, gram-negative, microaerophilic bacterium, which was established in 1982 by Robin Warren and Barry Marshall as the causative agent of gastritis and peptic ulcer [1,2], a discovery that revolutionized gastroenterology. Before Warren and Marshall, the human stomach was believed to be a sterile area. Today, *H. pylori *is recognized as the most common cause of gastritis, which in turn leads to the development of more gastrointestinal complications such as peptic and duodenal ulcers. Additionally, the organism is classified as a class 1 carcinogen because of its causal relationship to gastric adenocarcinoma, one of the world's deadliest cancers [3,4].

**Figure 1:**
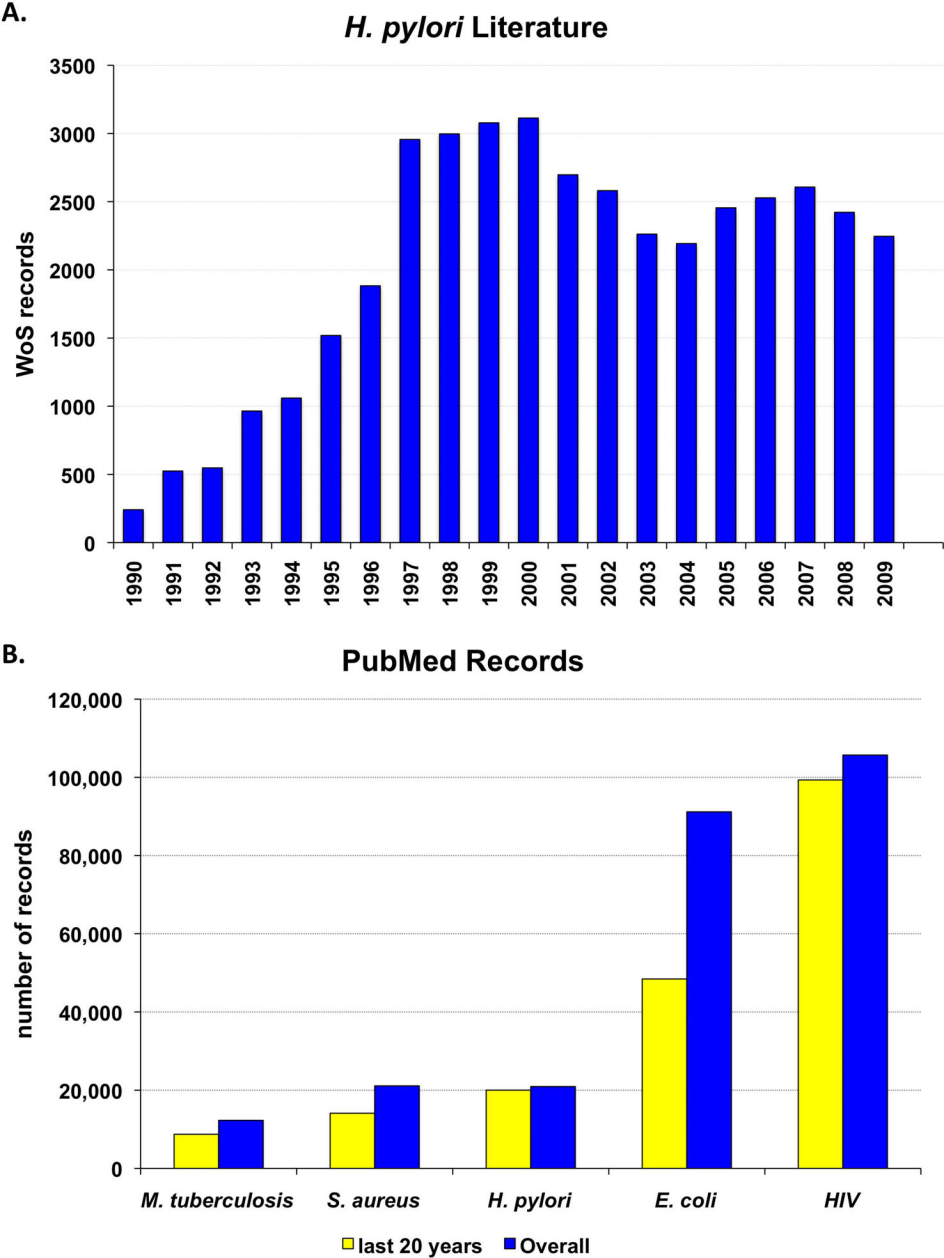
***A. H. pylori*-related articles published between 1989 and 2009**. Data are collected from search results on ISI Web of Science (WoS, URL: http://www.isiwebofknowledge.com) with "Helicobacter pylori OR H. pylori" as search string. **B. Example infectious agents in literature**. The PubMed literature database (URL: http://www.ncbi.nlm.nih.gov/pubmed) was searched for articles whose titles include the words: *Mycobacterium tuberculosis *(*M. tuberculosis*), *Staphylococcus aureus *(*S. aureus*), *Helicobacter pylori *(*H. pylori*), *Escherichia coli *(*E. coli*), and the human immunodeficiency virus (HIV). Although *H. pylori *is a recently discovered pathogen, it has more records in literature than well-established pathogens such as *S. aureus *and *M. tuberculosis *but is exceeded by HIV and *E. coli*, the latter being possibly the most cited bacterium and the most commonly used organism in the laboratory.

The previously underestimated clinical relevance of this rediscovered spiral bacterium quickly enticed microbiologists, epidemiologists, infectious disease specialists, and veterinarians to explore its physiology, genetics, epidemiology, and transmission. Such scientific activity was translated into more than 40,000 scientific articles about *H. pylori *in the past 20 years (Fig. 1A). During these 20 years, the number of *H. pylori*-related articles in PubMed http://www.ncbi.nlm.nih.gov/pubmed became comparable to the number of articles about well-established pathogens such as *Staphylococcus aureus *and *Mycobacterium tuberculosis *(Fig. 1B).

The growing attention given to *H. pylori *by academics and clinicians is not surprising since this pathogen colonizes more than half of the world's inhabitants [5], with an evident geographic variation in its epidemiology. This geographic variation is believed to be largely socioeconomically driven on both global and local scales. Other factors have also been reported to influence the incidence and prevalence of *H. pylori*, such as age, gender, genetic predisposition, ethnicity, educational level, and sanitation. Yet, the remarkably unequal burden of *H. pylori*-associated diseases on poorer communities and countries is the focus of this review article.

## Epidemiology of *H. pylori *Infection

### Prevalence of *H. pylori *infection

*H. pylori *is one of the most common bacterial infectious agents; it inhabits the stomachs of more than half of the world's population [5]. The prevalence of infection seems to mostly depend on the rate of acquisition (see below), but also on the rate of loss of infection [6] and the length of the persistence period between acquisition and loss [7]. Based on these factors, *H. pylori *prevalence differs from one country to another and may differ between different ethnic, social, or age groups within the same country [6,8-11].

Globally, the prevalence of *H. pylori *infection in developing countries is markedly higher than that in developed countries [12-16]. Moreover, the acquisition of *H. pylori *seems to occur at higher rates in developing countries [7,9]. A plethora of studies reported and emphasized these differences within and between countries (Table 1).

**Table 1:** Prevalence of *H. pylori *infection in different populations of the world.

Country	N studied cases	Age range (years)	Pre-valence	**Ref**.
**Developing Countries^1^:**				
**Bangladesh**	181	20-44	**92%**	[95]

**Brazil**				
- rural	40 (children)	< 20	**77.5%**	- rural
	164 (adults)	20-90	**84.7%**	[96]
- urban	363	> 20	**63.4%**	[97]
- poor urban community	204	18-80	**80%**	[98]

**Colombia **(rural)	684	2-9	**69%**	[99]

**China**				
- Southern China	1727	N/A	**44.2%**	[9]
- Hong Kong	397	36-65	**58.6%**	[100]
- Changle of Fujan	1456		**80.4%**	[100]

**Egypt**				
- Alexandria (northern)	169 mothers	N/A	**88%**	
	169 children	< 1	**13%**	
		1.5	**25%**	[101]
- Cairo (central)	52	< 6	**33%**	
	56	> 6	**60%**	[102]
- Assiut (southern)	urban	N/A	**87%**	
	rural	N/A	**40%**	[103]
- poor urban area	schoolchildren	N/A	**72.4%**	[104]

**India**	238	3-70	**79%**	[13]

**Mexico**	11605	20-90	**66%**	[61]

**Nepal **(rural)	1142	4-93	**56.8%**	[69]
	407	2 mo-12 yr	**48%**	[105]

**Peru**	104	0-17	**50%**	[70]

**Russia**	213	20-75	**88%**	[106]
- St. Petersburg 1995	307	2-19	**44%**	[18,106]
- St. Petersburg 2005	370	2-19	**13%**	[18]

**Saudi Arabia**	557	5-10	**40%**	[107]
		> 20	**70%**	

**Taiwan**	823	1-40+	**54%**	[78]

**Developed Countries^1^:**				
**Australia **(urban Melbourne)	273	19-47	**23%**	[32]

**Denmark**	3589	30-60	**25.9%**	[108]

**Germany **(western)	260	18-61	**39.2%**	[60]

**Israel **(rural)	377	30-90	**72%**	[109]

**Japan**	4361	19-69	**30%**	[110]

**Netherlands**	254 (employees)	11-89	**27.2%**	[111]

**New Zealand**	579 workers:	40-64	**56%**	
- Europeans	190		**35.8%**	
- Maori	195		**57.4%**	
- Pacific Islanders	194		**73.2%**	[64]

**Spain**	332	> 18	**43%**	[112]
- mountain	178^2^		**54%**	
- coastal	154		**30%**	

**South Korea**	161	20-75	**75%**	[113]

**Switzerland**	176 natives	10-20	**7.3%**	[81]
	20 immigrants		**30%**	

**United Kingdom**				
- England	267 (healthy)	> 18	**41%**	[114]
	467 (all males)	18-65	**37.5%**	[115]
- Northern Ireland	4742	12-64	**50.5%**	[116]
- South Wales	1796	45-59	**70%**	[117]

**United States**				
- South Carolina	938 army recruits	17-26	**26%**	
	324 blacks		**44%**	
	47 Hispanics		**38%**	
	536 whites		**14%**	[66]
- California	556	20-39	**27%**	[67]
- Texas	246 blacks	15-80	**70%**	
	239 whites		**34%**	[59]

Examples of differences in *H. pylori *prevalence within and between countries, representing the developing and developed world.

1. The classification into developing or developed countries was retrieved from the United Nation Development Programme's Human Development Reports (URL: http://hdr.undp.org/en)

2. The number reported in the article was 179, which is likely a typographical error.

N/A: Data not available or not applicable

### Incidence of *H. pylori *infection

The geographic differences in *H. pylori *prevalence have been attributed to the differential rate of acquisition of the bacterium during the first years of life [5,6,9,17,18]. In southern China, for example, the prevalence of *H. pylori *infection was shown to be significantly higher among Chinese subjects than that among Australians, a difference that was associated with the rate of acquisition of *H. pylori *under the age of ten years [9,11].

Acquisition of *H. pylori *is decreasing in developed countries at a faster rate than in developing countries, likely because of the faster improvement in hygiene practices in the developed world [5,19]. Moreover, infection during childhood in developed countries is not frequent [20-23]. In the United States, for example, the incidence of infection among children younger than five years is less than 5%, and only about 10% of the population is infected by adolescence [20,21]. By contrast, the incidence of *H. pylori *infection in the developing world is higher and occurs at younger age [22,24]. By five years of age, about 50% of children in developing countries are already infected [12,24], and the infection rates in adults can reach 90% or higher (Table 1).

Pounder and Ng classified the world into two groups according to the incidence of *H. pylori *infection [7]. Group One consisted of countries where the majority of children become infected with *H. pylori *during childhood, while chronic infection continues during adult life. These are mostly developing countries, e.g., Algeria, Nepal, South Africa, Saudi Arabia, Thailand, and Vietnam. In Group Two, mostly comprising developed countries, only a minority of children becomes infected during childhood, but the prevalence of infection rises with age during adulthood. Examples of Group Two countries are England, Finland, France, Japan, and the United States of America [7]. However, Pounder and Ng concluded their synthesis with an interesting question. Do the age-dependent prevalence data reflect that people in Group Two have more incidence of infection at older ages, or do the data rather reflect that the incidence of infection is declining in newer generations, which implies that the infected adults had been actually infected in their childhood [7]? This question was effectively answered later on, as longitudinal studies confirmed the birth cohort effect in the United States [6] and Russia [18], for example.

## Transmission of *H. pylori *infection

### I. Direct transmission [For a detailed review, see Ref. [11]]

The mode of transmission of *H. pylori *is one of the most controversial areas in the study of this pathogen. Ingestion of the bacteria, which is the most likely portal of entry, may occur by one or a combination of three means: oral-oral, gastro-oral, or fecal-oral, but determining a dominant route is not an easy task. While culturing *H. pylori *from the gastric secretion is possible, its isolation from stool or the oral cavity is difficult because either location is known for its diverse, abundant resident microbiota. Many members of this microbiota outgrow *H. pylori*, masking its colonies if present, unless a discriminating selective culture medium is used [11].

### 1) Oral-oral transmission

Although *H. pylori *was suggested as a member of the oral microbiota, independent from the stomach's infection status [25], and although the prevalence of *H. pylori *infection among dentists or dental workers is not higher than in others [26,27], the mouth is still being considered as a candidate reservoir for *H. pylori*, and oral-oral transmission is regarded as a plausible route of *H. pylori *transmission. Recent studies reported that exposure to persons with *H. pylori*-induced gastroenteritis is a risk factor for new infection [28], while experiments with rhesus macaques supported the hypothesis that oral-oral transmission is the most likely route of transmission [29]. This mode of transmission can be potentiated by specific eating habits, such as the premastication of food by mothers before feeding children in some African countries [30], and the use of chopsticks and communal eating in some immigrant Chinese communities [31,32], although the chopstick hypothesis has been challenged [33].

Detection of *H. pylori*-specific DNA from the oral cavity was reported [e.g., [34-36]], and even though isolating these bacteria from the oral cavity is difficult because of the presence of fast-growing microbiota, strains, identical to those isolated from the stomachs of the same patients, were successfully cultured [37-39]. On the other hand, several other studies failed to detect *H. pylori*-specific DNA from the oral cavities of *H. pylori*-positive patients [e.g., [40,41]].

### 2) Gastro-oral transmission

Since the human stomach is the primary niche of *H. pylori*, it is reasonable to suggest a direct gastro-oral route of transmission mediated by refluxed gastric juice [11,42]. This hypothesis is supported by several studies in which *H. pylori *was detected in the gastric juice [33,43-46] as well as the vomitus of infected subjects, and even from the air sampled during the vomiting process [47]. Additionally, a high rate of active *H. pylori *infection was detected by the ^13^C urea breath test in siblings of *H. pylori*-infected vomiting children [42].

The gastro-oral hypothesis also explains earlier reports of epidemic gastritis in subjects undergoing repeated gastric secretory studies [48,49], as well as observations of higher prevalence of *H. pylori *in gastroenterologists performing endoscopy [26].

### 3) Fecal-oral transmission

*H. pylori *is sensitive to the bile's bactericidal effect, so theoretically, and under normal conditions, passage of viable *H. pylori *through the intestine and its detection in stool are unlikely [11,50]; yet, some studies suggest that passage of viable *H. pylori *through the intestine could be verified [e.g., [51]]. While *H. pylori*-specific DNA was successfully detected in the fecal samples from as few as 10% to as many as 90% of subjects with known *H. pylori *infection [52-55], detection of *H. pylori*-specific DNA is obviously not a sufficient evidence for bacterial viability [11].

After several attempts for culturing *H. pylori *from fecal samples failed, viable bacteria were successfully isolated from one adult and 23 children in Gambia [56], and 12 adults in the United Kingdom [57]. Nevertheless, attempts to reproduce these results using similar techniques failed, which led to the suggestion that the malnourishment of Gambian children and their short fecal transit time are the reasons behind the exceptional isolation of *H. pylori *from fecal samples [58]. In support of the latter hypothesis, Parsonnet and colleagues showed that inducing diarrhea with a cathartic made culturing *H. pylori *from stool samples possible, also suggesting that gastrointestinal tract illnesses might increase *H. pylori *transmissibility [47].

### II. Indirect transmission

Environmental or animal reservoirs were investigated as sources of *H. pylori *infection. Food, animals, and water sources have been suggested as reservoirs outside the human gastrointestinal tract, and *H. pylori *or its DNA was detected in each of these sources (Table 2). However, there is no definitive evidence that they are natural or primary vehicles of transmission.

**Table 2:** *H. pylori *reservoirs.

Hypothesis	Evidence/Example studies	**Ref**.
**Food**		
- Contaminated food prepared under unhygienic conditions is a probable mechanism for transmission.	A positive correlation was reported between prevalence of infection and consumption of food from street vendors in Peru.	[70]
- The daily amount of raw vegetables is a risk factor, which possibly implies a role for water too.	In the Colombian Andes, frequent consumption of raw vegetables was associated with likelihood of infection.	[99]
- Sheep and cow milk can be vehicles for transmission.	See below (under Animals)	[118,119]

**Animals**		
- Several animal species were suggested as *H. pylori *reservoirs.	*H. pylori *was isolated from:	
	- pigtailed monkeys	[120]
	- rhesus monkeys	[121]
	- cats	[122]
	- sheep	[123]
	- cockroaches	[124]
	- houseflies (but the housefly hypothesis was challenged)	[125,126]
- Working with animals may increase risk, and animal-to-human transmission is possible.	*H. pylori *was suggested as zoonotic, occupational infection to meat and abattoir workers.	[127,128]
	In Colombian Andes, children who had contact with sheep had higher prevalence odds.	[99]
	Dore *et al*. stated that animals, especially sheep and dogs, could transmit *H. pylori *to humans (shepherds) in Sardinia.	[129]
- *H. pylori *can be recovered from animal products.	*H. pylori *was recovered from sheep and cow milk.	[118,119]
- Experimental animal colonization is possible.	Bacillary forms of *H. pylori *were used to colonize germ-free piglets.	[130]

**Water**		
- Water contamination is a risk factor for *H. pylori *infection.	Many reports, mostly from developing countries, suggested contaminated water sources as risk factors. Examples include studies performed in Peru, Chile, and Kazakhstan.	[105,131,132]
- Water from running, municipal, and underground sources, as well as wastewater has been suggested as reservoir for *H. pylori*.	- Municipal water	[105]
	- Well water	[133]
	- Running water	[99,134]
	- Wastewater	[135]
	- *H. pylori *even survives in chlorinated water.	[136]
- *H. pylori *proteins and DNA can be detected in water.	- by immunological methods	[137]
	- by PCR	[133,138]
	- by hybridization methods	[139]
- Viable *H. pylori *can be detected and isolated from water.	- as individual cells	[140]
	- associated with biofilm	[141,142]
	- cultured	[135]

Examples of studies on different environmental sources of *H. pylori *and their role in *H. pylori *transmission.

## Genetic and Environmental Determinants of *H. pylori *Epidemiology

### Age

The effect of age on the prevalence of *H. pylori *is one of the best-documented and least disputed aspects of *H. pylori *epidemiology. A positive correlation between age and prevalence has been reported in both developed and developing countries [for example, [8,59-61]]. Consistently, the prevalence of infection was found to be higher in adults than that in children, and this pattern has been interpreted to partly reflect a birth cohort phenomenon caused by a higher incidence in the past due to poor living conditions and sanitation [18,35,62,63].

A comprehensive longitudinal study undertaken by Fujisawa *et al*. evaluated changes in the seroepidemiological pattern of *H. pylori *in a group of Japanese people over a 20-year period [63]. Sera were collected from 1015 subjects, and the overall prevalence of *H. pylori*-specific antibodies was 72.7% in 1974, 54.6% in 1984, and 39.3% in 1994. The prevalence of *H. pylori *was found to be positively correlated with age, suggesting that there was a clear cohort-shift in the seroepidemiological pattern of *H. pylori *during the 20 years studied [63].

### Ethnic and genetic predisposition

Marked differences in *H. pylori *seroprevalence have been observed and reported among various ethnic and racial groups [10,14,64]. For example, in Malaysia, the increased risk of *H. pylori *infection in Chinese and Indians was suggested as an inherent ethnic genetic predisposition [10]. In New Zealand, ethnicity was suggested as a risk factor among different groups in the populations. *H. pylori *infection was most prevalent in Pacific Islanders, intermediate in Maori, and least prevalent in Europeans. Even after the adjustment of confounding factors, such as age and socioeconomic status, ethnicity remained a significant covariate [64].

By contrast, in a study conducted in USA, the prevalence of *H. pylori *infection was almost identical between Hispanic and African Americans, but significantly higher than that among Caucasians. However, ethnicity was ruled out as a major factor and the observed variance was attributed to socioeconomic conditions [8]. Finally, a study of monozygotic and dizygotic twins suggested also that genetic factors might have some influence on the incidence of *H. pylori *infection [65].

### Gender

An excess of *H. pylori *prevalence in one gender versus the other has been reported [66-68]; for instance, Woodward and colleagues observed a higher prevalence of *H. pylori *in men than in women [68]. Others found no gender-related difference in the prevalence of *H. pylori *infection [10,64,69,70]. A more recent, more comprehensive meta-analysis of large, population-based studies concluded a male predominance of *H. pylori*-related diseases in adults but not in children [71].

### Interfamilial relations

Many studies reported an influence of interfamilial relation on the spreading of *H. pylori *infection and highlighted adult-child transmission [72-74]. Konno and coworkers suggested mother-to-child transmission as the single most probable cause of the interfamilial spreading of *H. pylori *infection after a five-year follow-up study. Among 44 children enrolled in that study, five children acquired *H. pylori *infection, and their bacterial isolates exhibited DNA fingerprinting patterns identical to those of their mothers [74]. Family size has also been shown to positively affect *H. pylori *infection incidence; the relative risk of infection has been shown to increase according to the number of children per household [60,75].

Additionally, spouse-to-spouse transmission was suggested [76,77]. Georgopoulos *et al*. found a significant number of couples infected with indistinguishable strains of *H. pylori *[76].

Finally, infected children were also proposed as a source of infection for parents or siblings [60,78]. However, a case-control study performed in Bangladesh found no difference in infection rates between parents of infected and non-infected infants, and concluded that in communities with high prevalence of *H. pylori *infection, interfamilial transmission might be masked by other environmental factors [79]

### Socioeconomic factors

Socioeconomic status was reported as one of the most important factors affecting the spreading of *H. pylori *infection [8,14,80]. In particular, the high age-specific prevalence of *H. pylori *infection in developing countries has been attributed to low socioeconomic level [13]. By contrast, the lower prevalence of *H. pylori *infection in developed countries may be a result of higher socioeconomic status. The overall prevalence of *H. pylori *among Swiss adolescents was found to be 9.7%. While this prevalence is among the lowest in Europe, further analysis indicated that subjects from foreign countries had higher rates of infection (30%) than natives (7.3%). This significant difference was largely attributed to higher living standards among natives [81].

In USA, Malaty *et al*. classified children into five social classes. The prevalence of the infection was 82% in the lowest class, 52% in the two middle classes, and 11% in the two high classes, demonstrating an inverse correlation between *H. pylori *prevalence and socioeconomic status [72].

Obviously, socioeconomic status is not restricted to income and social class but takes in consideration other factors, including living standards, sanitation, urbanization, and educational level [11]. Combined, these factors are likely to increase the risk for infectious diseases in general.

Educational level, in particular, has been used as a marker of socioeconomic status and has been considered as one of the important determinants of *H. pylori *prevalence in both developed [82] and developing countries [61]. Rosenstock *et al*. found that the short duration of schooling beside low socioeconomic status increases the likelihood of *H. pylori *infection in Denmark [82].

### Crowding index (Density of living)

Household crowding, sharing a bed, and increasing household contact have been identified as risk factors of *H. pylori *infection [9,61,83,84]. In a large community-based study, Torres and colleagues stated that density of living conditions is a prime determinant in the acquisition of *H. pylori *[61]. In childhood, crowded living conditions affect current *H. pylori *status, and the number of children in the present household increases the risk of infection for the adult family members [60].

## Conclusion

### The verdict: Is *H. pylori *a poverty-associated disease?

As with most infectious and non-infectious diseases, no one factor can be singled out as the major determinant of *H. pylori *incidence and prevalence. However, there is credible evidence that poverty-associated factors are major players.

**Figure 2:**
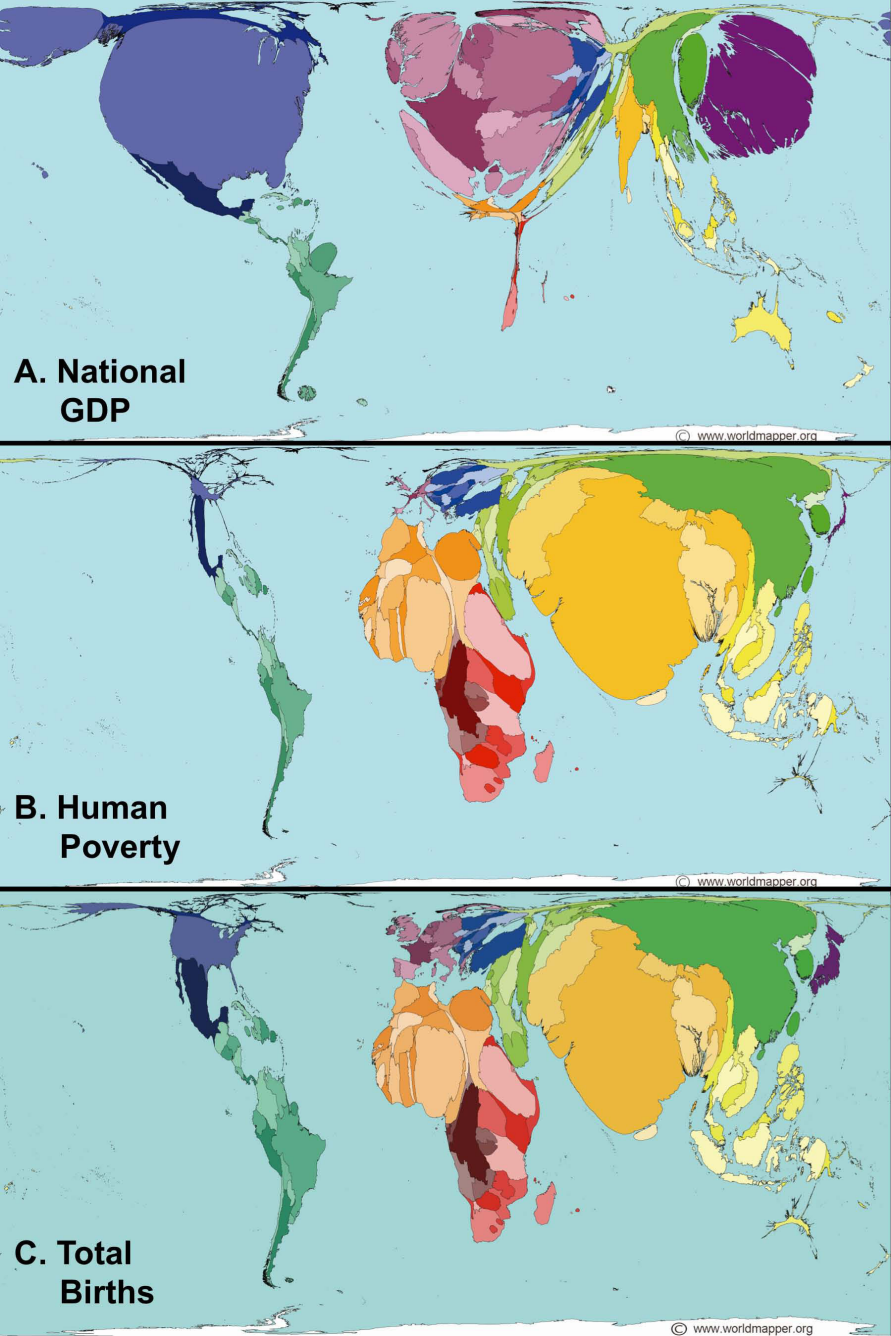
**Cartograms showing the unequal distribution of (A) wealth, (B) human poverty indices, and (C) total births around the globe**. The cartograms, or map projections, were obtained from URL: http://www.worldmapper.org with permission (^© ^Copyright SASI Group, University of Sheffield; and Mark Newman, University of Michigan). They had been generated by a diffusion-based method [91] and were included in the Worldmapper project [92-94].

The studies reviewed in this article show some evident differences in epidemiology between developing and developed countries, notably among children. However, we do not think that *H. pylori *prevalence is directly correlated with a country's overall wealth inasmuch as human poverty is not necessarily dependent on a country's gross domestic product (compare Fig. 2A with Fig. 2B). Instead, the effects of poverty on *H. pylori *infection are more pronounced between different communities, often located within the same country or region, but separated based on sanitation, overall hygiene, and standards of living. A good example is given in a recent study, conducted over 10 years, which showed that improved standards of living in Russia have substantially reduced *H. pylori *transmission [18].

### Outlook: Changing climate + changing demography + changing economy = redrawing the global map of *H. pylori *epidemiology

**"Out of every 100 persons added to the population in the coming decade, 97 will live in developing countries." Hania Zlotnik, 2005 **[85] (Hania Zlotnik is the director of the Population Division, Department of Economic and Social Affairs, United Nations Secretariat)

The last question we address in this review article is about the future of *H. pylori *epidemiology. The foreseeable future, unfortunately, does not seem very promising for the developing countries. Although *H. pylori *infection may eventually disappear from high-income countries even without intervention, as suggested by mathematical modeling [86], its prevalence is paradoxically expected to rise in low-income countries and communities [87]. The changing climate is expected to change the world's demography, resources, and clean water availability [88]. Combined, these factors have direct impact on living standards and hygiene, and are thus not likely to slow down the rate of *H. pylori *acquisition in developing countries [89]. If we add to these factors the unequal population growth (Fig. 2C), the uneven economic growth, and the rise in life expectancy all over the world, the picture gets dimmer. A likely scenario is that this combination of factors will translate into higher incidence of *H. pylori *infection in children, who, according to Hania Zlotnik, the director of the Department of Economic and Social Affairs-United Nations Population Division, will mostly be born and live in developing countries [85]. This higher incidence in children will lead to a rise rather than fall of *H. pylori*'s worldwide prevalence, and consequently to higher risk of gastric cancer especially in the elder population of the developing world (which are expected to live longer than they do today without necessarily being healthier).

Because of this serious risk, researchers should combine efforts and seek efficient methods to prevent *H. pylori*-associated diseases [87] and perhaps to eradicate this chronic colonizer of half of the human race [90].

## Competing interests

The authors declare that they have no competing interests regarding the publication of this review article.

## Authors' contributions

MMK surveyed and reviewed the literature, and drafted the paper; RRS collected recent articles and participated in writing the final version of the paper; RKA analyzed the collected data, reviewed the socioeconomic literature, and wrote the paper in its final format. All authors read and approved the final manuscript.

## Authors' information

Mohammed Mahdy Khalifa is a graduate student at the Faculty of Pharmacy, Cairo University, Cairo, Egypt. He received his master's degree in microbiology and immunology in 2009. Radwa Raed Sharaf is an undergraduate student at the Faculty of Pharmacy, Cairo University, Cairo, Egypt. Ramy Karam Aziz is currently a lecturer of microbiology and immunology at the Faculty of Pharmacy, Cairo University, Cairo, Egypt and an adjunct faculty member at San Diego State University, San Diego, CA, USA.
